# Personality traits and the risk for Parkinson disease: a prospective study

**DOI:** 10.1007/s10654-015-0062-1

**Published:** 2015-07-01

**Authors:** Johanna Sieurin, Petter Gustavsson, Caroline Elise Weibull, Adina Leiah Feldman, Giselle Maria Petzinger, Margaret Gatz, Nancy Lee Pedersen, Karin Wirdefeldt

**Affiliations:** Department of Medical Epidemiology and Biostatistics, Karolinska Institutet, Box 281, 171 77 Stockholm, Sweden; Department of Clinical Neuroscience, Karolinska Institutet, Nobels väg 9, 171 65 Stockholm, Sweden; MRC Epidemiology Unit, University of Cambridge, Box 285, Cambridge Biomedical Campus, Cambridge, CB2 0QQ UK; Department of Neurology, University of Southern California, 1333 San Pablo Street, MCA 243 9153 HSC, Los Angeles, CA 90033 USA; Department of Psychology, University of Southern California, 3620 South McClintock Ave, Los Angeles, CA 90089-1061 USA

**Keywords:** Parkinson disease, Personality, Introversion, Neuroticism, Cohort study

## Abstract

In this study, we explored the association between the personality traits, neuroticism and introversion, and risk of Parkinson disease (PD). A population-based cohort study was conducted using questionnaire data from the Swedish Twin Registry for twins born 1926–1958 (n > 29,000). Personality traits were assessed in 1973 by a short form of Eysenck’s Personality Inventory. The cohort was followed from 1974 to 2012 through Swedish patient and cause of death registers for PD ascertainment. Cox proportional hazards regression was used to estimate subsequent risk of PD, adjusting for attained age, sex and smoking. A mediation analysis was performed to further explore the role of smoking in the relationship between personality trait and PD. Confounding by familial factors was explored using a within-pair analysis. During a mean follow-up time of 36.8 years, 197 incident PD cases were identified. Both neuroticism and introversion were associated with an increased risk of PD after adjustment. Smoking was a significant mediator in the relationship between personality traits and PD that partly accounted for the effect of introversion, whereas it acted as a suppressor for the effect of neuroticism on PD risk. In the within-pair analyses, associations for neuroticism and introversion were attenuated. In conclusion, our study provides evidence that neuroticism is associated with an increased risk of PD that is in part suppressed by smoking. There was a weak association between introversion and PD and this effect was at least partly mediated through smoking. The observed effects may partly be explained by familial factors shared by twins.

## Introduction

Parkinson disease (PD) has long been related to a specific personality type, sometimes referred to as the ‘parkinsonian personality’, typically characterized as rigid, introverted, cautious and conservative. During the last decades it has been discussed whether there are specific personality traits that precede PD onset and, if so, whether these traits are risk factors or represent early manifestations of PD [1]. Results from retrospective case–control studies have suggested that traits such as anxiety proneness, introversion, low novelty seeking, cautiousness and rigidity precede PD onset [2–5], while others found no association with any of the big five personality traits, including extraversion and neuroticism [6]. More recent cohort studies have associated trait anxiety and neuroticism [7, 8] but not introversion [9] with an increased PD risk. Thus, the results have been inconsistent and the traits analyzed, as well as methods of personality assessment, have varied among different studies. Most studies have been retrospective and different time periods have been used, ranging from 5 to 40 years before PD diagnosis, and sometimes undefined. Hence, a personality related to PD cannot be clearly defined and whether the traits are risk factors or prodromal symptoms of PD is not certain.

PD is a slowly progressing disorder, in which the neurodegeneration starts years before the first movement symptoms appear. The preclinical period has commonly been estimated at 5–6 years based on autopsy studies of nigrostriatal dopamine loss [10]. However, other studies suggest a longer preclinical period, starting with Lewy body pathology in non-dopaminergic neurons outside substantia nigra [11]. Epidemiological studies also suggest a longer preclinical period, characterized by non-motor manifestations including autonomic dysfunction, olfactory impairment and neuropsychiatric symptoms such as depression and anxiety [10]. In order to determine the relation between personality and PD, a prospective design with a long follow-up time is of importance, but few such studies have been performed.

In this study, we tested the hypothesis that high levels of neuroticism and introversion are associated with an increased risk for PD in a cohort with up to 39 years of follow-up. We also explored smoking as a mediator and possible confounding by familial factors.

## Methods

### Study population

The study population included same-sexed twins in the Swedish Twin Registry (STR) [12], born in Sweden between 1926 and 1958, who responded to a mailed questionnaire in 1973 covering demographic, medical and life-style factors, as well as personality. All twins in this STR cohort who were residing in Sweden and not previously diagnosed with PD at the start of follow-up (January 1st 1974) were eligible to enter the study cohort (36,409). In total, 436 persons were excluded due to migration during follow-up or because it was not possible to link their data. Among the remaining population, 6121 twins did not provide sufficient questionnaire data on the personality assessment and were excluded. This resulted in a study population of 29,852 individuals.

The study was approved by the Regional Ethical Vetting Board in Stockholm, Sweden and the Institutional Review Board of the University of Southern California, USA.

### Parkinson’s disease ascertainment

Incident cases of PD were identified through cross-linkage of the STR with the National Patient Register (NPR) [13] and the Cause of Death Register (CDR) [14]. The NPR was initiated in 1964 and contains information about discharge records from hospitals in Sweden. In 1976, NPR covered more than 50 % of the in-patient care; and since 1987, the coverage of the in-patient care is complete [15]. Since 2001, the NPR also includes outpatient records from hospital-based clinics. Each record contains one primary diagnosis and contributory diagnoses coded according to the International Classification of Diseases (ICD). The CDR has covered all Swedish counties since 1961 and contains information from death records, including underlying cause of death and contributory causes coded according to the ICD.

Cases were defined as PD if they had a primary PD diagnosis in the NPR or CDR. ICD codes used for PD ascertainment were: 342.00 (ICD-8); 332.0 (ICD-9); G20 (ICD-10). Date of ascertainment was defined as the first date of any PD diagnosis in the NPR, or date of death in the CDR for those cases only identified at death.

### Personality assessment

Personality data were collected as part of the questionnaire sent out in 1973, when the respondents were between 15 and 48 years old. This questionnaire included a short form of the Eysenck Personality Inventory (EPI-Q) [16], comprising 18 items, 9 each from the Neuroticism and Introversion scales [17]. The response format was yes/no and item response was translated to a value of 0 or 1 and a summed score (0–9) was calculated for each scale. To be included in the study, individuals had to respond to at least 6 items on either trait. For respondents with 1–3 items missing on a trait, a score (0–9) was imputed based on the distribution of the provided responses.

### Smoking status

Due to previous research showing the importance of smoking as a protective factor in PD [18], smoking status was included as a covariate in the adjusted models and a mediational analysis was performed. Information was taken from the questionnaire in 1973 and for those who participated in the Screening Across the Lifespan Twin Study (SALT), structured computer-assisted telephone interviews conducted in 1998–2002. Smoking status was categorized as ever versus never smoker. Among those with an assessed smoking status 70 % had data from both occasions, 29 % only had data from the questionnaire in 1973, and 1 % only had data from SALT.

### Statistical methods

The associations between continuous and binary variables were analyzed by logistic regression and relationships between two continuous variables were assessed by linear regression. Associations with PD were analyzed using time-to-event methods with attained age as underlying time scale. Risk time, expressed in terms of person-years, was accumulated from date of study entry (January 1st 1974) until first recorded PD diagnosis, death or when follow-up ended (December 31st 2010), whichever came first. Incidence rates were calculated as events divided by person-time at risk reported per 100,000 person-years with 95 % confidence intervals (CI) assuming a Poisson distribution.

Neuroticism and introversion were treated both as continuous variables and as categorical variables divided into quartiles based on the distribution of the data. The quartile of lowest scores was used as reference group. We also tested for a linear trend across quartiles. Cox proportional hazards regression, yielding hazard ratios (HR) with 95 % CI was used to evaluate possible associations between personality traits and PD incidence. These were interpreted as measures of relative risk. Sex and smoking status were included as covariates in the adjusted models. We also explored sex and smoking as effect modifiers by including interaction terms with these variables. A robust sandwich estimator of the standard errors was used to account for the dependence between observations in the twin data.

To investigate the role of smoking as a mediator in the relationship between personality trait and PD, a mediation analysis was performed according to the approach suggested by Lange et al. [19]. This analysis decomposes the total effect of an exposure into estimates of the so-called natural direct and indirect effects using nested counterfactuals. See Lange et al. [19, Appendix 2] for a description of this method and an example SAS code. The natural direct and indirect (i.e. mediated through smoking) effects were calculated adjusting for sex as a baseline confounder and using attained age as the underlying time scale. The 95 % CIs were calculated using a bootstrap method with 1000 replications.

To test for confounding by familial factors shared within twin pairs, we further conducted a conditional Cox regression model with twin pair as the stratum variable, thereby fixing an individual baseline hazard within each pair of twins while at the same time allowing it to vary between twin pairs [20]. Only complete twin pairs discordant for personality and PD diagnosis contribute to the within pair analyses. An attenuation of any observed effect would suggest that familial factors contribute to the association, while persistence of the effect would suggest an independent effect of personality on PD incidence.

The assumption of proportional hazards was assessed using the Thernneau and Grambach test [21] of the Schoenfeld residuals. No evidence of non-proportionality was found. Data analyses were performed using SAS (9.3) for Windows.

## Results

The study population consisted of 29,852 twins with an even sex distribution. Mean age was 30.2 years (SD 9.2) at baseline and 67.0 years (SD 9.6) at the end of follow-up. During a mean follow-up time of 36.8 years (SD 6.1) we identified 197 incident cases of PD. Mean age at PD ascertainment was 67.6 years (SD 8.9). As expected, the incidence of PD increased with age and was higher in men and in never smokers (Table 1).

**Table 1:** Frequencies and incidence rates (IR) of PD with 95 % CI by sex, attained age, smoking status, neuroticism and introversion

	N (%)	PD cases	IR^a^	(95 % CI)
Total	29,852 (100)	197	17.9	(15.6–20.6)
Sex				
Men	14,212 (48)	104	20.1	(16.6–24.4)
Women	15,640 (52)	93	16.0	(13.0–19.6)
Attained age				
<60	7519 (25)	36	4.2	(3.0–5.9)
60–70	11,271 (38)	71	42.1	(33.3–53.1)
>70	11,062 (37)	90	116.0	(94.3–143.0)
Smoking				
Never	11,228 (38)	99	23.6	(19.4–28.8)
Ever	18,006 (60)	95	14.4	(11.8–17.6)
Missing	618 (2)	3		
Neuroticism				
Continuous	29,802 (>99)	197		
Quartile 1	5340 (18)	28	14.2	(9.8–20.6)
Quartile 2	10,262 (34)	73	19.2	(15.3–24.2)
Quartile 3	7197 (24)	55	20.7	(15.9–26.6)
Quartile 4	7003 (23)	41	16.1	(11.8–21.8)
Missing	50 (<1)	0		
Introversion				
Continuous	29,766 (>99)	195		
Quartile 1	7594 (25)	39	13.9	(10.2–19.1)
Quartile 2	9100 (30)	50	14.9	(11.3–19.7)
Quartile 3	8036 (27)	64	21.6	(16.9–27.6)
Quartile 4	5036 (17)	42	22.6	(16.7–30.6)
Missing	86 (<1)	2		

^a^Incidence rate (IR) per 100,000 person years

Neuroticism was associated with an increased risk of PD when treated as a categorical variable, both in the crude model adjusted only for attained age and after further adjustment for sex and smoking (Table 2). In the crude model, there was a significantly increased risk of PD in the third quartile compared to the first quartile with the lowest neuroticism scores. The association between neuroticism and PD risk was strengthened in the multivariable-adjusted model, and there were significant associations with PD comparing both the second and third quartile to the first. The relationship between neuroticism and PD was pronounced in women, with HRs above two both for the third and fourth quartile compared to the first, a significant trend across neuroticism quartiles and a significant HR when neuroticism was treated as a continuous variable, whereas there were no significant associations in men. However, the interaction term between sex and neuroticism was not significant (*p* = 0.11) (Table 2).

**Table 2:** Hazard ratios for incidence of PD with 95 % CIs by personality scores in the entire cohort and by sex

Personality trait	Crude		Multi-adjusted		Multi-adjusted, by sex			
					Men		Women	
	N	HR (95 % CI)	n	HR^b^ (95 % CI)	N	HR^c^ (95 % CI)	N	HR^c^ (95 % CI)
Neuroticism								
Continuous	197	1.03 (0.97–1.08)	194	1.05 (0.99–1.11)	101	1.00 (0.91–1.09)	93	1.09 (1.01–1.18)
Quartile 1	28	1.00	28	1.00	21	1.00	7	1.00
Quartile 2	73	1.49 (0.97–2.31)	73	1.56 (1.01–2.41)	43	1.39 (0.82–2.34)	30	2.12 (0.93–4.81)
Quartile 3	55	1.68 (1.06–2.64)	52	1.74 (1.10–2.77)	25	1.50 (0.84–2.68)	27	2.49 (1.09–5.72)
Quartile 4	41	1.37 (0.84–2.23)	41	1.59 (0.98–2.59)	12	1.00 (0.47–2.11)	29	2.68 (1.17–6.10)
*p* trend^a^		0.20		0.06		0.74		0.02
Introversion								
Continuous	195	1.06 (1.00–1.13)	192	1.07 (1.01–1.14)	99	1.06 (0.97–1.15)	93	1.09 (1.00–1.18)
Quartile 1	39	1.00	38	1.00	26	1.00	12	1.00
Quartile 2	50	1.03 (0.68–1.57)	49	1.07 (0.70–1.63)	28	1.03 (0.61–1.76)	21	1.17 (0.58–2.38)
Quartile 3	64	1.38 (0.93–2.06)	63	1.43 (0.95–2.13)	27	1.12 (0.65–1.91)	36	1.92 (1.00–3.70)
Quartile 4	42	1.32 (0.86–2.03)	42	1.41 (0.91–2.17)	18	1.37 (0.76–2.46)	24	1.59 (0.80–3.17)
*p* trend^a^		0.07		0.04		0.32		0.06

All models have attained age as underlying time scale

*HR* hazard ratio, *CI* confidence interval

^a^Trend across quartiles

^b^Adjusted for sex and smoking status

^c^Adjusted for sex, smoking status and an interaction term between personality trait and sex

There were significant associations between introversion and PD risk, both in the linear model and for the trend across quartiles adjusting for attained age, sex and smoking (Table 2). There were no significant associations between introversion and PD in the crude model, although the tendency was similar. The HRs were higher in women than in men, but none were significant, including the interaction term (*p* = 0.67) (Table 2).

Neuroticism was associated with significantly increased odds of smoking [OR 1.12 (95 % CI 1.11–1.13)] and introversion was associated with significantly decreased odds of smoking [OR 0.91 (0.90–0.92)]. As shown before [18], smoking had a protective effect on PD risk and could therefore be a potential mediator in the relationship between personality trait and PD—acting as a suppressor for the effect of neuroticism. A mediation analysis was performed to further explore the role of smoking. The association between neuroticism and PD was strengthened when looking at the direct effects and all HRs were significant (Table 3). The indirect effect of neuroticism through smoking was weakly protective for PD, yet significant. The direct effect of introversion on PD was lower than the total effect, due to the positive indirect effect going through smoking, although the HRs for the indirect effects were close to unity (Table 3).

**Table 3:** Mediation analysis: hazard ratios for the total effects, natural direct effects and natural indirect effects (through smoking) with 95 % CIs, adjusted for sex and with attained age as underlying time scale

Personality trait	Total effects		Natural direct effects		Natural indirect effects	
	HR	(95 % CI)	HR	(95 % CI)	HR	(95 % CI)
Neuroticism						
Quartile 1	1		1		1	
Quartile 2	1.53	(0.99–2.36)	1.59	(1.04–2.60)	0.97	(0.95–0.99)
Quartile 3	1.68	(1.06–2.66)	1.76	(1.11–2.84)	0.96	(0.92–0.99)
Quartile 4	1.50	(0.92–2.43)	1.62	(1.02–2.71)	0.93	(0.87–0.98)
Introversion						
Quartile 1	1		1		1	
Quartile 2	1.07	(0.70–1.64)	1.06	(0.70–1.66)	1.01	(1.00–1.02)
Quartile 3	1.45	(0.97–2.17)	1.42	(0.95–2.18)	1.02	(1.00–1.04)
Quartile 4	1.46	(0.94–2.25)	1.36	(0.88–2.17)	1.04	(1.01–1.08)

*HR* hazard ratio, *CI* confidence interval

Next, we performed within-pair analyses using twin pairs discordant on personality to investigate whether the observed associations were confounded by familial factors. The HRs in the within-pair analyses dropped compared to previous analyses, both for neuroticism and introversion (Table 4). Although the confidence intervals overlapped, this attenuation of the effect is an indication of confounding by genetic or early life environmental factors.

**Table 4:** Hazard ratios for PD with 95 % CIs by personality traits, adjusted for familial factors shared within twin pairs

Personality trait	n	HR^b^ (95 % CI)
Neuroticism		
Continuous	163	1.02 (0.92–1.15)
Quartile 1	23	1.00
Quartile 2	69	1.31 (0.64–2.68)
Quartile 3	40	1.55 (0.67–3.57)
Quartile 4	31	1.17 (0.50–2.74)
*p* value^a^		0.73
Introversion		
Continuous	162	1.04 (0.92–1.19)
Quartile 1	35	1.00
Quartile 2	43	1.06 (0.53–2.13)
Quartile 3	48	1.06 (0.50–2.24)
Quartile 4	36	1.27 (0.56–2.89)
*p* value^a^		0.59

*HR* hazard ratio, *CI* confidence intervals

^a^Trend across quartiles

^b^Within-pair analysis of twins by stratified Cox model with attained age as underlying time scale. Adjusted for smoking status and matched on sex and familial factors shared within twin pairs

We also analyzed the four combinations of low and high (median split) levels of neuroticism and introversion, with low neuroticism/low introversion as the reference group. The results suggested that the combination of high neuroticism and high introversion was associated with the highest risk of PD in an additive manner (results not shown).

Finally, we adjusted for education and number of hospital visits during the follow up period. Education (mandatory vs. higher education) was considered to be a proxy for socioeconomic status. The total number of recorded hospital visits for all causes other than PD was counted for each individual, and considered to be a proxy for help-seeking behavior that might be related to personality traits and possibly influence the probability of being ascertained with PD. We found a significant association between neuroticism and increased number of hospital visits for all causes (β = 13.4, *p* < 0.0001), whereas introversion was not associated with number of hospital visits (β = 1.6, *p* = 0.10). None of these factors markedly changed the estimates (were not statistical confounders) and were thus not included in the final models.

## Discussion

Using a large population-based cohort of Swedish twins with a follow-up time of up to 39 years, we found that both neuroticism and introversion are associated with an increased risk for PD. The effect of these traits was more evident in women, especially for neuroticism, although there were no significant interactions with sex. Smoking was an important mediator that partly accounted for the effect of introversion whereas it acted as a suppressor for the effect of neuroticism. This is the largest study to date and the first prospective cohort study in twins.

In line with previous studies [7, 8], our findings indicate that neuroticism, which is defined as an increased tendency to emotional reactivity and instability [22] is a specific risk factor for PD. People with high neuroticism scores both experience stressful events more often and have an increased vulnerability to environmental stress [23]. Stress has been hypothesized as risk factor for PD [24, 25], and has received some support from experimental studies, but not by epidemiological data [26, 27]. A vulnerability to stress and chronic activation of the hypothalamic–pituitary–adrenal (HPA) axis may be mediating factors, partly explaining the relationship between neuroticism and PD. This is the first prospective study identifying an association between introversion and risk of PD. Although some retrospective case–control studies considering introversion a relatively short time (5 years) before PD diagnosis have found an association [3, 4], other studies report null associations [6, 9]. Given the long follow-up time (6–39 years for cases) in the present study and assessment of personality traits relatively early in life, it is unlikely that these traits represent prodromal symptoms. However, since personality is only measured once, and the prodromal period for PD may be very long, a prodrome cannot be ruled out.

Smoking is a well-known protective factor for PD [28], as was also evident in our data. We found a positive association between neuroticism and smoking and an inverse association between introversion and smoking. Theoretically, this would lead to a deflation of the total effect of neuroticism and an inflation of the total effect of introversion, which was confirmed by the mediation analysis. The main interest of the current study was the direct effects of personality traits on PD risk. Based on the results from the multi-adjusted models and the ‘natural direct effects’ from the mediation analysis, our general interpretation is that there is an association between neuroticism and PD that could not be explained by smoking (or other variables adjusted for) and that there is a weak association between introversion and PD, that partly could be explained by smoking status. It is important to note, however, that no statistical methods can distinguish between confounding and mediation; that decision has to be based on background knowledge. Although it cannot be excluded that smoking induces changes in personality, which would mean that smoking is a confounder, we believe that smoking more likely is a mediator in the relationship between personality traits and PD.

It has been suggested that the protective effect of smoking on PD risk could be explained by either confounding by personality or reverse causation due to pre-clinical personality changes that make PD susceptible individuals less prone to smoking [29]. However, we found that personality traits and smoking had separate effects on PD risk, at least for neuroticism.

Another potential explanation for the observed association between personality and the risk of PD is confounding by familial factors, such as shared genes or familial environment. For instance, it has been reported that relatives of PD patients have an increased risk for anxiety and depressive disorders [30]. Therefore, it might be plausible that some third variable shared by families influences both personality development and risk of PD. The influence of confounding by familial factors shared within twin pairs can be studied by comparing the association between the personality traits and PD in the initial cohort analyses with the results from the within-pair analyses. As the HR was smaller in the within-pair analysis, this finding indicates that the results may partly be explained by familial confounding. Unfortunately, the sample size was markedly reduced in the within-pair analyses, limiting firm conclusions.

The main strengths of the present study are the prospective design, the size of the cohort, the long follow-up time and use of a population-based sample. Another advantage is that the exposure was measured relatively early in life using of a standardized instrument for assessing neuroticism and introversion. Further, the use of a twin cohort is a novel approach in this context, which allowed us to explore the effect of familial factors. Finally, we were able to adjust for several important factors.

A limitation of this study is that the study population was relatively young; the mean age at exit of the study was 67 years. Therefore, the incidence rate for PD observed in this cohort was relatively low. The use of health registers for identification of cases is only an approximation of incident cases of PD. However, a previous validation study of PD diagnoses in the NPR and CDR indicates that the accuracy and sensitivity is generally good, but misclassification between PD and other parkinsonian disorders occurs [31]. Restricting the definition of cases to primary diagnoses improves the accuracy, although at the cost of reduced sensitivity. Misclassification of diagnosis is likely non-differential, and would most probably introduce bias towards the null. Further, the results were similar both when PD cases were identified through primary diagnosis or both primary and secondary diagnoses.

Neuroticism is associated with hypochondrical symptoms [32, 33] and people with high scores of neuroticism may be more prone to seek medical help. This could differentially influence the probability of being ascertained with PD and lead to biased findings. To deal with this issue we counted and adjusted for the total number of hospital visits for causes other than PD during the follow-up. We found a strong association between neuroticism and increased number of hospital visits, whereas introversion was unrelated to number of hospital visits. Importantly however, adjustment for hospital visits did not influence the association with PD, neither for neuroticism nor introversion.

In summary, our study indicates that both neuroticism and introversion increase the risk for PD. The tendency is similar in both sexes, although pronounced in women. Smoking acts as a suppressor in the relationship between neuroticism and PD, whereas it partly accounts for the association between introversion and PD. The observed associations might partly be confounded by familial factors, but this remains to be further explored.
